# Development of Adamantane-Conjugated TLR7/8 Agonists for Supramolecular Delivery and Cancer Immunotherapy

**DOI:** 10.7150/thno.35434

**Published:** 2019-11-26

**Authors:** Christopher B. Rodell, Maaz S. Ahmed, Christopher S. Garris, Mikael J. Pittet, Ralph Weissleder

**Affiliations:** 1 Center for Systems Biology, Massachusetts General Hospital, Boston, MA 02114, USA; 2 Graduate Program in Immunology, Harvard Medical School, Boston, MA 02115, USA; 3 Department of Radiology, Massachusetts General Hospital, Boston, MA 02114, USA; 4 Department of Systems Biology, Harvard Medical School, Boston, MA 02115, USA

**Keywords:** nanoparticle, cyclodextrin, drug screening, drug delivery, immunotherapy, macrophage

## Abstract

Tumor-associated macrophages (TAMs) are often abundant in solid cancers, assuming an immunosuppressive (M2-like) phenotype which supports tumor growth and immune escape. Recent methods have focused on identification of means (e.g., drugs, nanomaterials) that polarize TAMs to a tumor suppressive (M1-like) phenotype; however, reducing the systemic side effects of these therapies and enabling their delivery to TAMs has remained a challenge.

**Methods:** Here, we develop R848-Ad, an adamantane-modified derivative of the toll-like receptor (TLR) 7/8 agonist resiquimod (R848) through iterative drug screening against reporter cell lines. The adamantane undergoes guest-host interaction with cyclodextrin nanoparticles (CDNPs), enabling drug loading under aqueous conditions and TAM-targeted drug delivery. Therapeutic efficacy and systemic side effects were examined in a murine MC38 cancer model.

**Results:** R848-Ad retained macrophage polarizing activity through agonization of TLR7/8, and the adamantane moiety improved drug affinity for the CDNP. In preclinical studies, nanoformulated R848-Ad resulted in a drastic reduction in measurable systemic effects (loss of body weight) relative to similarly formulated R848 alone while arresting tumor growth.

**Conclusions:** The findings demonstrate the ability of strong nanoparticle-drug interactions to limit systemic toxicity of TLR agonists while simultaneously maintaining therapeutic efficacy.

## Introduction

Myeloid cells have attracted attention as therapeutic targets in cancer. These cells include tumor-associated macrophages (TAMs), which accumulate at high density in a broad range of tumors. These cells commonly assume a pro-angiogenic and immunosuppressive (“M2-like”) phenotype *in vivo*, supporting continued tumor growth 1. TAMs can also negatively impact the efficacy of anticancer drugs, including checkpoint blockade immunotherapies; their abundance in tumors is therefore associated with altered patient survival 2-4. TAMs thereby contribute to a tumor immune microenvironment (TIME) that supports accelerated tumor growth and resistance to therapy.

However, macrophage phenotypes are plastic, motivating the development of means to polarize these cells to an anti-tumorigenic and immunosupportive (“M1-like”) phenotype, bolstering their tumoricidal capacity. A number of pharmacological activators of TAM activity are under active investigation. These drugs include small molecule inhibitors of tyrosine kinases, histone deacetylases (HDACs), indoleamine 2,3-dioxygenase (IDO), and colony stimulating factor 1 receptor (CSF1R), amongst others 5, 6. In addition, antibodies directed against immune checkpoints expressed by myeloid cells (e.g., TIM-3) or self-recognition pathways (e.g., CD47/SIRPɑ axis) have been used to perpetuate anti-tumor TAM activity 7, 8. While many of these agents are proving effective through a number of direct and indirect mechanisms, activation of TAMs through established pathogen and danger-associated molecular pattern (PAMP/DAMP) pathways remains among the most potent means of myeloid activation 9, 10. These pathways include signaling through the endosomal toll-like receptors (TLRs) 7 and 8, which are highly potent activators of both innate and adaptive immunity that can be effectively agonized by small molecule drugs 11, 12.

Immune agonists are generally effective cancer immunotherapeutics; though, their clinical applicability is limited by systemic side effects. Indeed, several small-molecule TLR7/8 agonists have been clinically approved (e.g., imiquimod) or are under ongoing investigation. However, repeated systemic dosing of these drugs has been hindered by adverse events (headache, fever) that are dose-limiting or result in discontinuation of treatment, thereby hampering their clinical efficacy 13. To overcome this challenge, biomaterial-assisted delivery of immunostimulatory agents may be an effective means of localizing drug effects to the TIME. The development of local immunostimulatory implants has been recently explored, including the local delivery of drugs that promote local anti-tumor immune activity 14.

Importantly, targeting of TAMs through methods of systemic delivery would extend these therapies to include application in a metastatic setting or where direct tumoral access and surgical resection is not practical. Working toward this goal, we have previously reported on the development of β-cyclodextrin nanoparticles (CDNPs), which were used to encapsulate a potent TLR7/8 agonist (i.e., resiquimod, R848) through supramolecular guest-host association. Rapid uptake of the saccharide particles by immune cells enabled TAM repolarization and anti-tumor immune response, which synergized with the immune checkpoint inhibitor anti-PD1 5. However, the rapid release of R848 from CDNP remains a potential limiting factor in reducing systemic side effects.

In this study, we set out to simultaneously address the need to reduce systemic side effects and enable TAM-targeted delivery of immunostimulatory agents. We therefore performed a systematic search for chemical modifications of R848 that would improve the drug-nanoparticle association while retaining TLR7/8 agonization. Through iterative, systematic modifications of R848, we identified a derivative modified by adamantane through an aromatic linkage (R848-Ad) which retained TLR activation, was readily loaded into CDNPs through simple mixing under aqueous conditions, and which arrested tumor growth while minimizing side effects in mice. The approach represents a promising strategy to achieve functional reorientation of the TIME to an immune-active state through systemic drug delivery which targets activation of TAMs.

## Materials and Methods

**Materials.** Unless otherwise indicated, reagents were obtained from Sigma-Aldrich and used as received, water used was of MilliQ grade, and reagents where maintained under sterile conditions.

**Preparation of R848 derivatives**. Resiquimod was obtained from Selleckchem. The preparation and characterization of additional drug compounds is described in the Supplementary Methods. All drug compounds were prepared as 100 mM stock solutions in DMSO and stored at -20 °C until use.

**Nanoparticle synthesis.** Lysine cross linked cyclodextrin nanoparticles (CDNPs) were prepared following previously reported methods, and particles were fluorescently labeled by VivoTag 680 (VT680) where necessary for imaging studies 5. Succinyl-β-cyclodextrin (150 mg, 1.0 eq. succinylate), EDC (890 mg, 10.0 eq. to succinylate), and NHS (330 mg, 5.0 eq. to succinylate) were dissolved in 3.0 mL of 50 mM MES buffer at pH 6.0. After 30 min, *L*-lysine (42 mg, 0.5 eq. to succinylate) was dissolved in 1.5 mL MES buffer and added to the reaction which was stirred at room temperature overnight. The product was recovered by addition of 200 μL of brine and precipitation from excess cold ethanol. Following dissolution in water, the product was concentrated and repeatedly washed by water in centrifugal filters (Amicon; 10 kDa MWCO). Four separate batches were prepared and combined to afford the final product which was lyophilized, dissolved at 50 mg/mL in water, and stored at -20 °C.

**Cell models.** Cells were maintained in the indicated medium at 37 °C and 5% CO_2_ and regularly screened for contamination by mycoplasma. Reporter cell lines were obtained from InvivoGen, including HEK-Blue mTLR7 (hkb-mtlr7), HEK-Blue hTLR8 (hkb-htlr8), and THP1-Lucia NF-kB cells (thp1-nfkb). Cells were maintained following manufacturer's protocols; assays were performed at passage 5-8 for HEK-Blue mTLR7 cells and before passage 12 for HEK-Blue hTLR8 cells to maintain reporter activity. The MC38 mouse colon adenocarcinoma cell line was provided by M. Smyth (QIMR Berghofer Medical Research Institute) and maintained in Iscove's DMEM (Invitrogen) supplemented with 10% fetal calf serum (Atlanta Biologicals), 100 IU penicillin (Invitrogen), and 100 μg/mL streptomycin (Invitrogen).

Murine bone-marrow-derived macrophages (BMDMs) were isolated and derived as previously described 15. BMDMs were seeded at 0.5 × 10^6^ cells per well in 48-well plates (Corning) and maintained in Iscove's DMEM supplemented with 10% heat-inactivated fetal calf serum, 100 IU penicillin, 100 μg/mL streptomycin (Invitrogen) and 10 ng/mL recombinant murine M-CSF (PeproTech, 315-02). Tumor-associated macrophages (TAMs) were isolated 12 days after tumors were initiated in C57BL/6 mice by intradermal injection of 2 × 10^6^ MC38 cells suspended in 50 μL of PBS. Tissue was minced, incubated for 30 min in RPMI containing 0.2 mg/mL collagenase I (Worthington Biochemical), and passed through a 40 μm cell strainer. Red blood cells were lysed (ACK lysis buffer, Thermo Fisher Scientific), cells treated with Fc receptor blocking reagent (TruStain FcX anti-CD16/32 clone 93, BioLegend), and stained in PBS containing 0.5% BSA and 2 mM EDTA with fluorochrome labeled antibodies against mouse CD45 (30-F11, eBioscience) and F4/80 (BM8, BioLegend). 7-AAD (Sigma) was used as a viability dye. Macrophages (7AAD-CD45+ F4/80+) were sorted on a BD FACSAria. TAMs were plated at 10 × 10^3^ cells per well in 96-well plates (Corning) which were maintained and treated under conditions identical to BMDMs.

**Drug screening.** Cell proliferation was assessed by PrestoBlue (Fisher) following the manufacturer's protocols. Drug activity against HEK-Blue mTLR7, HEK-Blue hTLR8, and THP1-Lucia NF-kB cells was assessed following manufacturer's protocols for HEK-Blue (Invitrogen, hb-det2) and Quanti-Luc (Invitrogen, rep-qlc1) detection, respectively (N=4 wells per condition). Briefly, HEK-blue cells expressing either mTLR7 or hTLR8 were seeded 96-well plates at 0.25 × 10^6^ cells per well in HEK-Blue detection media containing the drugs of interest. After 16 hours, the absorbance at 662 nm was measured (Tecan, Spark) and is presented following background subtraction relative to non-treated control wells. THP1-Lucia cells were similarly treated, with seeding at a concentration of 0.5 × 10^6^ cells per well in RPMI 1640 supplemented with 10% fetal calf serum (Atlanta Biologicals), 100 IU penicillin (Invitrogen), 100 μg/mL streptomycin (Invitrogen), and 50 ng/mL recombinant human IFN-γ (PeproTech, 300-02). After 16 hours, conditioned media was combined with QUANTI-Luc detection media. Luminescence was measured (Tecan, Spark) and is presented as raw luminescence or fold change, relative to non-treated controls.

**Macrophage polarization.** Transcriptional analysis was performed after 24 h of drug treatment. BMDMs or TAMs were treated with drugs at a concentration of 1.0 nM to 10 μM. Treatment with media alone, IL4 (10 ng/mL; PeproTech, 214-14), or LPS (100 ng/mL; Sigma, L2630) and IFN-γ (50 ng/mL; PeproTech, 315-05) served as internal controls for M0, M2-like and M1-like transcription profiles, respectively. RNA was isolated (QIAGEN, 74106), subject to reverse transcription (Thermo Fisher, 4368814) and qPCR (Thermo Fisher, 44-445-57) for analysis of *hprt* (Thermo Fisher, Mm01545399_m1), *nos2* (Thermo Fisher, Mm00440502_m1), and *mrc1* (Thermo Fisher, Mm00485148_m1). Data are presented as the fold change (log2(∆∆CT)) in gene expression relative to *hprt* between treatment and M2-like control conditions.

**Characterization of guest-host interaction.** Guest-host interactions were examined by two-dimensional NMR spectroscopy and measurement of equilibrium binding affinity. For NMR, R848-Ad was combined with β-cyclodextrin, mixed overnight at room temperature, and lyophilized to afford a white powder which was re-dissolved in D_2_O to afford final concentrations of 10 mM β-cyclodextrin and 5 mM R848-Ad. The sample was filtered, degassed, and ROSEY spectra with solvent suppression collected on a Bruker AC-400 MHz spectrometer. Analysis of binding affinities for β-cyclodextrin was performed by standard competitive binding assays, described elsewhere 5, 16. Examination of R848-Ad solubilization by CDNP was performed by measurement of sample turbidity. R848-Ad was prepared as 2.5 mM in PBS at CDNP concentrations up to 5.0 %wt/vol. Absorbance at 365 nm was measured (Tecan, Spark) in optical bottom 96-well plates (Corning).

**CDNP drug loading and release.** Drug loading of nanoparticles by either R848 (R848@CDNP) or R848-Ad (R848-Ad@CDNP) was performed by dissolution the drugs into CDNP solutions. For preparation of a single dose (10 mg/kg R848-Ad; 0.2 mg/mouse), 3.725 μL of R848 or R848-Ad (100 mM in DMSO) was added to 100 μL of 5.0 %wt/vol CDNP in sterile saline and mixed overnight at room temperature. For R848-Ad control injections, 5.0 %wt/vol sulfobutylether-β-cyclodextrin (MedChemExpress) in saline was used to achieve drug solubility. As this procedure directly dissolve the drug into the CDNP without need for additional purification, quantitative drug loading (i.e., 100% loading efficiency) was assumed for all subsequent studies.

For release studies, formulations of R848@CDNP and R848-Ad@CDNP were prepared as described, having a final concentration of 5.0 mM drug and 2.5 %wt/v CDNP. Drug release was subsequently performed in an equilibrium dialysis setup (Bel-Art, H40317-0000; VWR, 470163-408) at 37 °C. At specified time points, the release buffer was removed from the cell and replaced with fresh buffer. The samples were lyophilized, reconstituted at 20x concentration in DMSO and concentration quantified by LCMS, measuring UV absorbance at 315 nm relative to standard curves. Data is presented following normalization to cumulative release of R848, N=3 samples per group.

**Nanoparticle characterization.** For both CDNP and R848-Ad@CDNP, particle size was calculated by dynamic light scattering (Malvern, Zetasizer APS) in PBS buffer at a concentration of 5 mg/mL. Samples were prepared for scanning electron microscopy by dilution to 100 μg/mL in water and freeze-drying on silica wafers. Pd/Pt sputter coated samples were imaged (Zeiss, Ultra Pulse), and size determined in by direct measurement in ImageJ (N=50 particles, ≥3 independent samples). Zeta potential was measured at a sample concentration of 100 μg/mL in 10 mM PBS immediately following instrument calibration to manufacturer standards (Malvern, Zetasizer ZS).

For examination of nanoparticle uptake, RAW264.7 cells were plated in 96-well plates (Ibidi) at 10 × 10^3^ cells/well. After 24 h, VT680 labeled CDNP was added (50 μg/ 350 mL) for 1 h. Fixed (4% paraformaldehyde, 30 min, 37 °C) cells were stained (nuclei: DAPI, Invitrogen; cell membrane: 5.0 μg/mL wheat germ agglutinin, Thermo Fisher; lysosome: anti-LAMP1 Alexa Fluor 488), washed, and imaged.

**Tumor growth models.** Animal studies were conducted in compliance with the National Institutes of Health guide for the care and use of Laboratory animals using female C57BL/6 mice (Jackson, 000664, 6-8 weeks of age). Protocols were approved by the Institutional Animal Care and Use Committees at Massachusetts General Hospital. Drug tolerance was assessed by examination of body weight in mice following administration of R848 or R848-Ad, formulated as described. Tumor growth was initiated in mice by intradermal injection of 2 × 106 MC38 cells in 50 μL of PBS. At 8 days, treatment cohorts were assigned with normalization of tumor size and body weight across groups. Mice were treated every third day by i.v. administration of CDNP (5.0 mg/mouse), R848-Ad or R848-Ad@CDNP formulated as described. Tumor growth was assessed by caliper measurement at set time points, and tumor volumes (V = (L*W^2^)/2) are reported.

**Statistical analysis.** Statistical analyses and line fitting were performed in GraphPad Prism 8. Data are presented as mean ± standard deviation for *in vitro* studies and as mean ± standard error for *in vivo* studies. For comparison of two groups, two tailed Student's t-test was used. For multiple comparisons, statistical significance was determined by one-way ANOVA with post hoc Tukey's HSD test. For temporal data, outliers were identified by Grubb's test and excluded. Comparison was performed by two-way repeated measures ANOVA with post hoc Fisher's LSD or, for groups of unequal size, by Friedman's Test with post hoc Dunn's test. Significance was assigned at P<0.05.

## Results and Discussion

**Development and screening of adamantane conjugated TLR7/8 agonists.** Toll-like receptors (TLRs) play an important role in activation of the immune system by alerting it to pathogen-associated molecular patterns (PAMPs), and small molecule agonist of these receptors have demonstrated success as immunotherapeutics 17. Specifically, imidazoquinolines are a potent family of readily modifiable compounds for TLR7/8 activation that includes R848 (a potent dual TLR7/8 agonist) 18-20. While acting as efficient macrophage polarizing agents in topical applications, the systemic administration of TLR agonists may be hampered by poor pharmacokinetics (rapid renal excretion) and pharmacodynamics (limited bioavailability in the tumor) 11.

To address these issues, differing approaches have been taken. For example, a series of lipophilic imidazoquinoline derivatives has been developed for localized injection within a tumor 21, 22. Alternatively, our group and others have investigated the nanoformulation of TLR7/8 agonists to enable systemic delivery 5, 23. Formulation by saccharide based materials, namely cyclodextrin nanoparticles (CDNPs), enables rapid uptake by TAMs. However, this method may be limited by the moderate affinity of existing TLR agonists for cyclodextrin. To improve cyclodextrin affinity, we sought to modify R848 through incorporation of adamantane (Ad) to increase the guest-host binding affinity. Many aromatic compounds, such as tryptophan, exhibit moderate affinity for β-cyclodextrin, with an equilibrium binding constant (K_eq_) on the order of 10^3^ M^-1^ 24-26. R848 exhibits similar properties, with a dissociation constant (K_D_) of 7.84 ± 0.51 mM^-1^ which may be improved by greater than 300-fold through conjugation with adamantane (K_D_ = 20.4 ± 1.33 μM^-1^). Conjugation of Ad to existing TLR agonists therefore represents a promising method to improve drug binding to cyclodextrin nanoparticles, improving their supramolecular delivery to TAMs.

**Figure 1:**
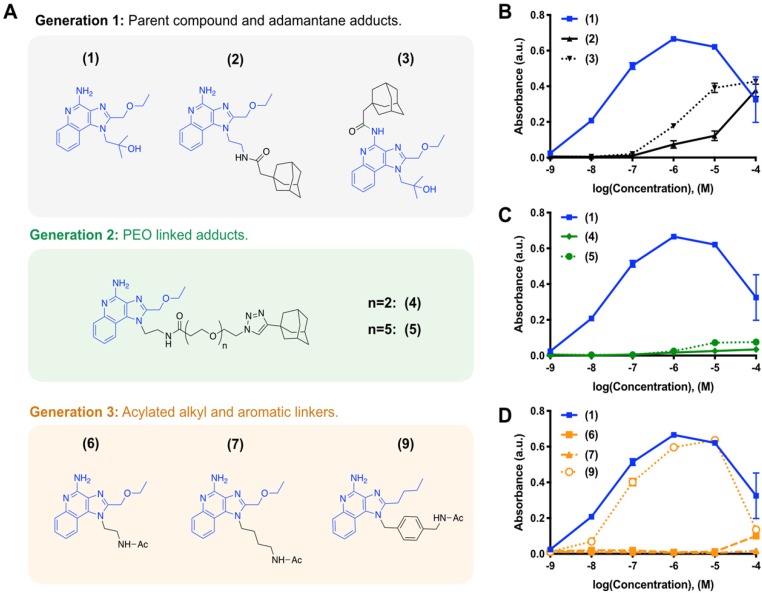
** Drug development through targeted library screening.** (**A**) Targeted library developed through iterative modification of compound **(1)**; the base structure is highlighted in blue throughout. (**B-C**) Concentration-dependent activity of compounds in Generation 1 (**B**, black), Generation 2 (**C**, green), and Generation 3 (**D**, orange) assayed against HEK-Blue mTLR7 cells. Results represent the mean ± s.d. of the absorbance at λ=662 nm; N=4.

We iteratively synthesized and tested a number of different R848 analogs (Figure 1) with the overarching goal of retaining TLR activation while incorporating adamantane functionalization at a position suitable for cyclodextrin association. Compounds were screened against HEK-Blue cells expressing the murine TLR7 receptor, where the SEAP reporter is under control of the IL12-p40 promoter to allow for rapid identification of compounds which are both specifically active against the TLR7 receptor and which promote IL12-p40 which is among the most significant indicators of both polarization and immunotherapy response 27, 28. Initially, we sought to modify the R848 structure directly through the addition of adamantane at either the aniline or accessible tetrabutoxy tail positions. These compounds (Generation 1, compounds **(2)** and **(3)**) were easily synthetically accessed; however, a dramatic decrease in activity was observed when compared to the parental compound, R848, **(1),** in treated cells (Figure 1B). Examination of the crystal structure (Figure S1) suggested the direct addition of adamantane may hinder drug interaction with the binding site, which would explain the reduced activity. Additionally, poor solubility was observed for both compounds **(2)** and **(3)**, likely contributing to the poor drug bioavailability. To address these issues, we next generated compounds that incorporated a poly(ethylene oxide) (PEO) linker between the core R848 structure and adamantane (Generation 2, compounds **(4)** and **(5)**). The addition of the PEO linker improved water solubility and removed the adamantane from the protein binding site (Figure S1), better enabling drug-protein interaction. However, these modifications result in a loss of drug activity (Figure 1C). As increased water solubility of was inversely correlated with drug activity, we developed a third generation of compounds with alkyl and aryl linkers (Generation 3, compounds **(6, 7** &** 9)**). Of these, **(9)** displayed highly potent drug activity (Figure 1D), comparable to **(1)**. This is in line with prior reports, which have investigated the development of polymer-linked TLR agonists via aromatic linkages 29. *In vitro* drug activity of **(9)** suggested the aryl linker as a handle by which further drug optimization might be accomplished.

**Figure 2:**
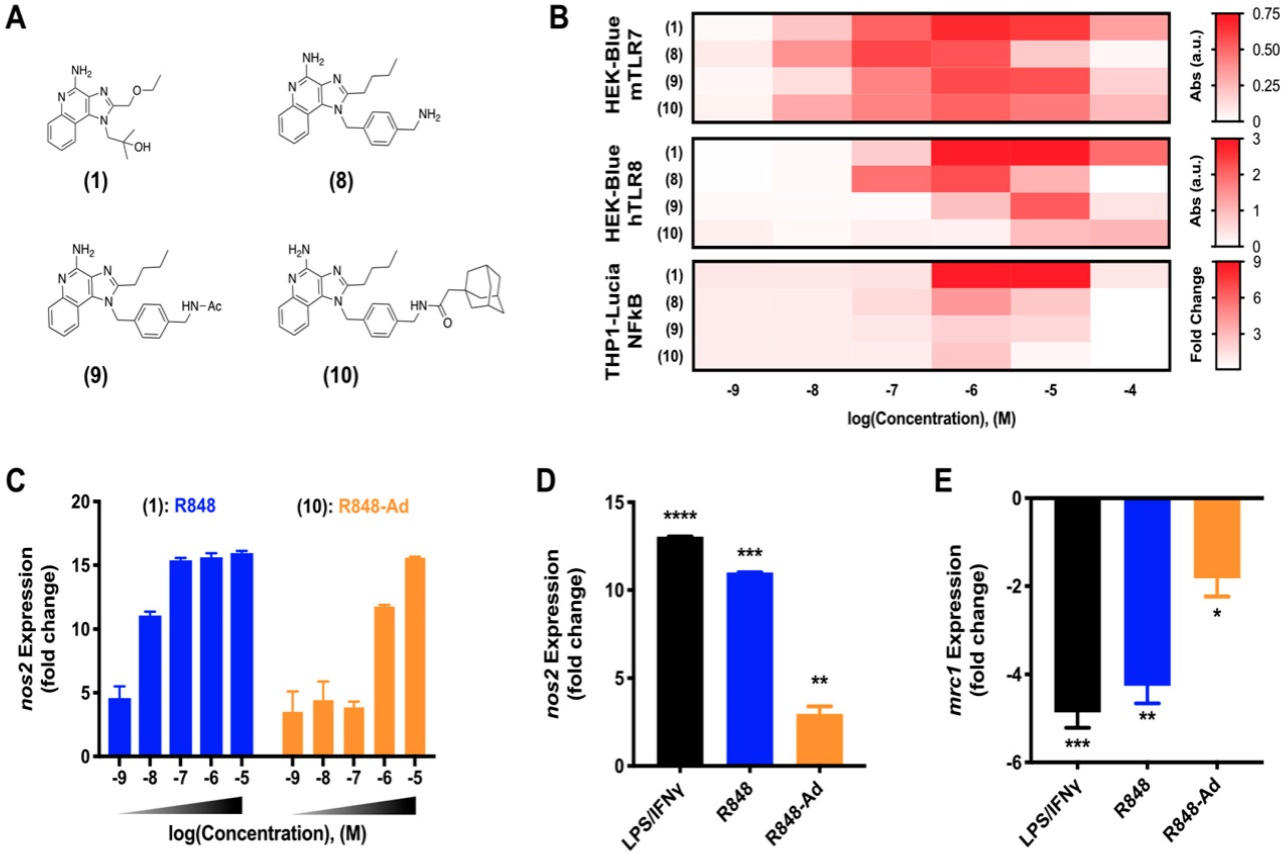
** R848-Ad maintains TLR7/8 activation potential and acts as a macrophage repolarizing agent.** (**A**) Compounds examined. (**B**). Concentration-dependent activity of compounds HEK-Blue mTLR7 (top), HEK-Blue hTLR8 (middle), and THP1-Lucia NFkB (bottom) reporter cell lines. Results represent the mean of N=4 independent repeats. (**C**) Expression of *nos2*, indicative of M1-like polarization, by bone marrow derived macrophages in response to drug treatment. Results are normalized to expression by untreated (M0) cells. Mean ± s.d.; N=3. (**D-E**) Repolarization of TAMs isolated from MC38 tumors. Expression of *nos2* (**D**) and *mrc1* (**E**) by TAMs following drug treatment by LPS/ IFN-γ, R848 (100 nM), or R848-Ad (100 nM). Results are normalized to untreated controls. Mean ± s.d.; N=3; ****p<0.0001, ***p<0.001, **p<0.01, *p<0.01 vs untreated controls.

**Macrophage activation through TLR7/8 agonization.** As discussed, activity of small molecule drugs may be altered by factors including both the drug solubility and docking site affinity. Having identified **(9)** as a candidate for continued in depth study of TLR7/8 activation, we next sought to examine the activity of derivatives including various modifications of the aromatic linker (Figure 2A). Modifications include the aminated aromatic linker **(8)**, the acylated aromatic linker **(9)**, and the adamantane adduct of interest **(10)**.

While TLR7 and TLR8 are considered to be redundant in some circumstances, there are differences between the murine and human receptor expression profiles and protein structures which influence their drug response. These differences culminate in a blunted response of human TLR7, and reports that murine TLR8 is a mute receptor 18, 30, 31. To ensure drug activity in both model murine systems and the relevant human case, drug activity was examined against key reporter cell lines 9. These included murine TLR7 and human TLR8 expressing cell lines, HEK-Blue mTLR7 and HEK-Blue hTLR8, respectively, in addition to the human THP1 (THP1-Lucia NFkB) reporter cell line. Outcomes for compounds **(1)** through **(9)** were qualitatively similar when screened against HEK-Blue hTLR8 and THP1 cells (Figure S2), corroborating outcomes from initial activity against mTLR7. While agonist activity of **(8-10)** was reduced by increasingly hydrophobic modifications across all cell lines, **(10)** retained moderate drug activity. Due to the drug activity and the successful incorporation of adamantane to bolster cyclodextrin binding, **(10)** was selected for further study in primary murine cells and is henceforth referred to as R848-Ad.

Reporter cell lines provide an optimized system, ideal for readily screening drug activity against an individual receptor. However, they do not fully recapitulate the complex signaling pathways of mature primary immune cells 9. To better examine the activity of R848-Ad in this context, we examined expression of canonical M1-like (*nos2*) and M2-like (*mrc1*) genes in bone marrow derived macrophages (BMDMs) as well as in TAMs harvested from mature MC38 tumors in C57BL/6 mice. In BMDMs (Figure 2C), both R848 and R848-Ad treated cells exhibited an increase in expression of *nos2;* the half-maximal effective concentration was increased (LogEC_50_ = -6.16 ± 0.13 M), relative to unmodified R848 (LogEC_50_ = -8.25 ± 0.57 M). TAMs isolated directly from MC38 tumors were responsive even to low doses (100 nM) of R848-Ad (Figure 2D,E), with increased expression of *nos2* and reduced expression of *mrc1*, relative to untreated controls. In sum, R848-Ad is an effective modulator of macrophage phenotype, including in macrophages derived in the complex *in vivo* tumor microenvironment. Drug activity proceeds through the relevant receptors in each species, including mTLR7 and hTLR8, resulting in NFkB signaling and a phenotypic switch in the hallmark M1-like and M2-like expression signatures.

**Figure 3:**
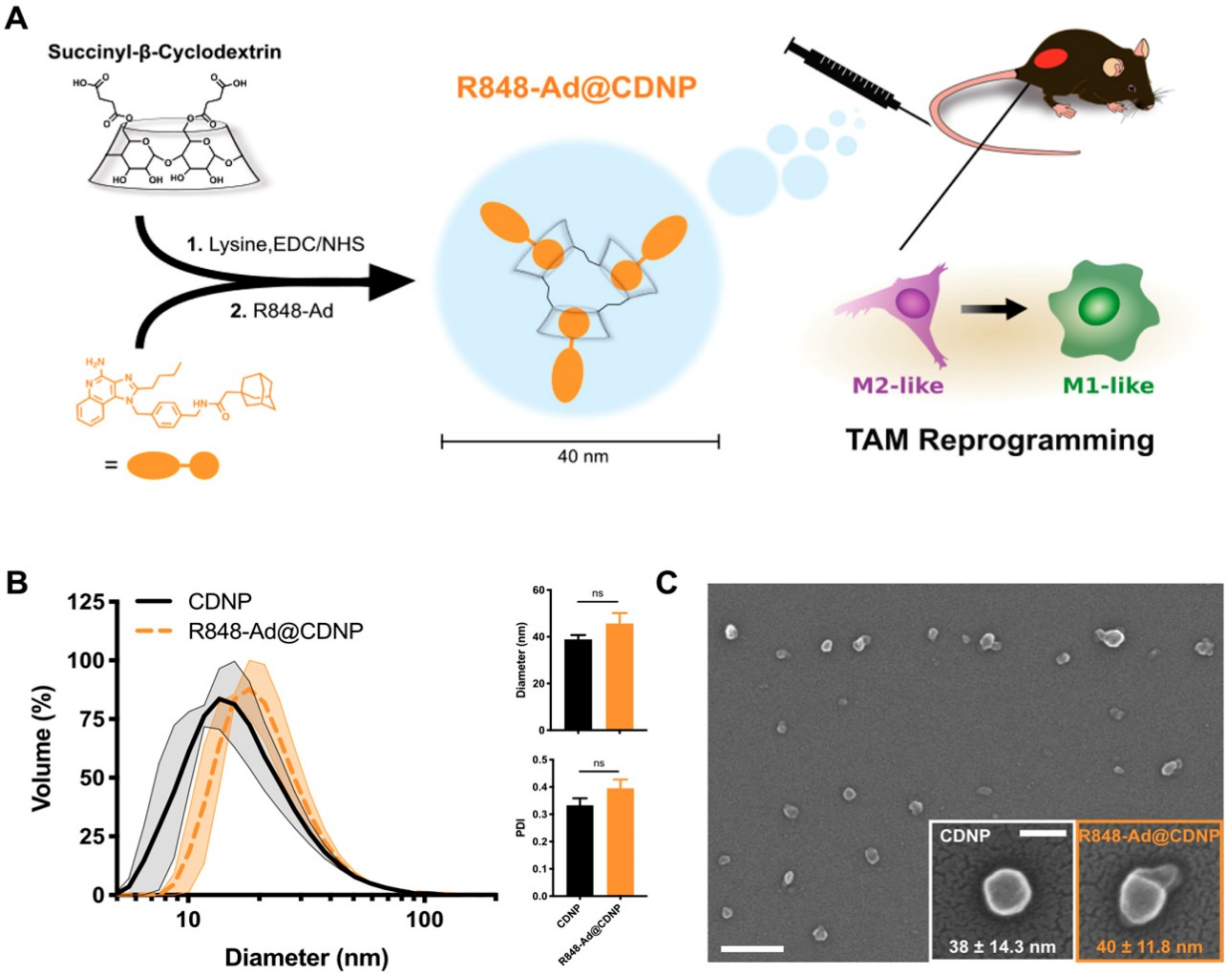
** Nanoparticle synthesis and characterization.** (**A**) Schematic overview of cyclodextrin nanoparticle (CDNP) preparation and drug loading. Succinyl-β-cyclodextrin was cross linked by *L*-lysine and subsequently loaded with R848-Ad through guest-host interaction. *In vivo*, drug laden nanoparticles (R848-Ad@CDNP) repolarize TAMs to an active (M1-like) phenotype. (**B**) Histogram of the nanoparticles' hydrodynamic diameter before (CDNP) and after (R848-Ad@CDNP) drug loading, measured by dynamic light scattering. Quantification of diameter and polydispersity index (PDI). N=3 independent replicates; ns = not significant. (**C**) Scanning electron microscopy images of R848-Ad@CDNP. Scale bar: 200 nm. Inset: higher resolution images of both CDNP and R848-Ad@CDNP and quantification of diameter. Mean ± s.d.; scale bar: 50 nm.

**Characterization of cyclodextrin nanoparticles and drug interaction.** The delivery of nanomaterials to TAMs is an effective method for achieving the targeted modulation of the TIME 6, 32, 33, including through the delivery of encapsulated cytokines (e.g., IL12), or owing to the material properties themselves 34-36. For macrophage targeted delivery, polyglucose-based particles are particularly useful due to their rapid uptake which has motivated their prior use as imaging agents 37-39. By employing β-cyclodextrin as the base material, our lab has recently developed polyglucose nanoparticles which are suitable for macrophage targeting and which act as a drug delivery vehicle for hydrophobic drugs 5, 40. The unique cyclodextrin nanoparticles (CDNPs) exhibit a high affinity for macrophage uptake due to the polyglucose content while providing a guest-host interaction between the encapsulated drug (guest) and the macrocycle cavity (host), enabling drug delivery to TAMs for their therapeutic repolarization (Figure 3A).

CDNPs were prepared by crosslinking succinyl-β-cyclodextrin with *L*-lysine under buffered aqueous reaction conditions. The reaction proceeded overnight after addition of 0.5:1 molar equivalents *L*-lysine to succinyl groups. Nanoparticles were collected with excellent yield (>90%) by precipitation from iced ethanol and subsequent centrifugal concentration, resulting in a particle size of 39 ± 1.8 nm, as determined by DLS (Figure 3B). Following loading with R848-Ad by mixing under aqueous conditions, there was minimal change in particle size (46 ± 4.5 nm, DLS) or polydispersity index (PDI). Both empty (CDNP) and drug-loaded (R848-AD@CDNP) particles exhibited a roughly spherical shape when examined by scanning electron microscopy (Figure 3C), and aggregation of insoluble drug was not observed. The zeta potential of both CDNP and R848-Ad@CDNP, were slightly negative (-7.77 ± 1.34 mV and -6.61 ± 1.03 mV, respectively), as is desirable for avoidance of hepatic uptake and rapid macrophage uptake* in vivo* 41. In sum, CDNPs were readily prepared and loaded with R848-Ad under aqueous conditions. The process yielded drug-laden particles above the renal excretion threshold which are small enough to achieve tissue penetration and rapid macrophage uptake 42. We have previously observed that these properties result in excellent biodistribution to the tumor, with as much as 4.1 ± 1.15% of the injected dose being locally delivered with a concurrent TAM-associated cellular distribution 5. Additionally, these materials are rapidly uptaken by phagocytic cells (Figure S3), perpetuating delivery of the encapsulated drugs to their target receptors, which are endosome-associated.

**Figure 4:**
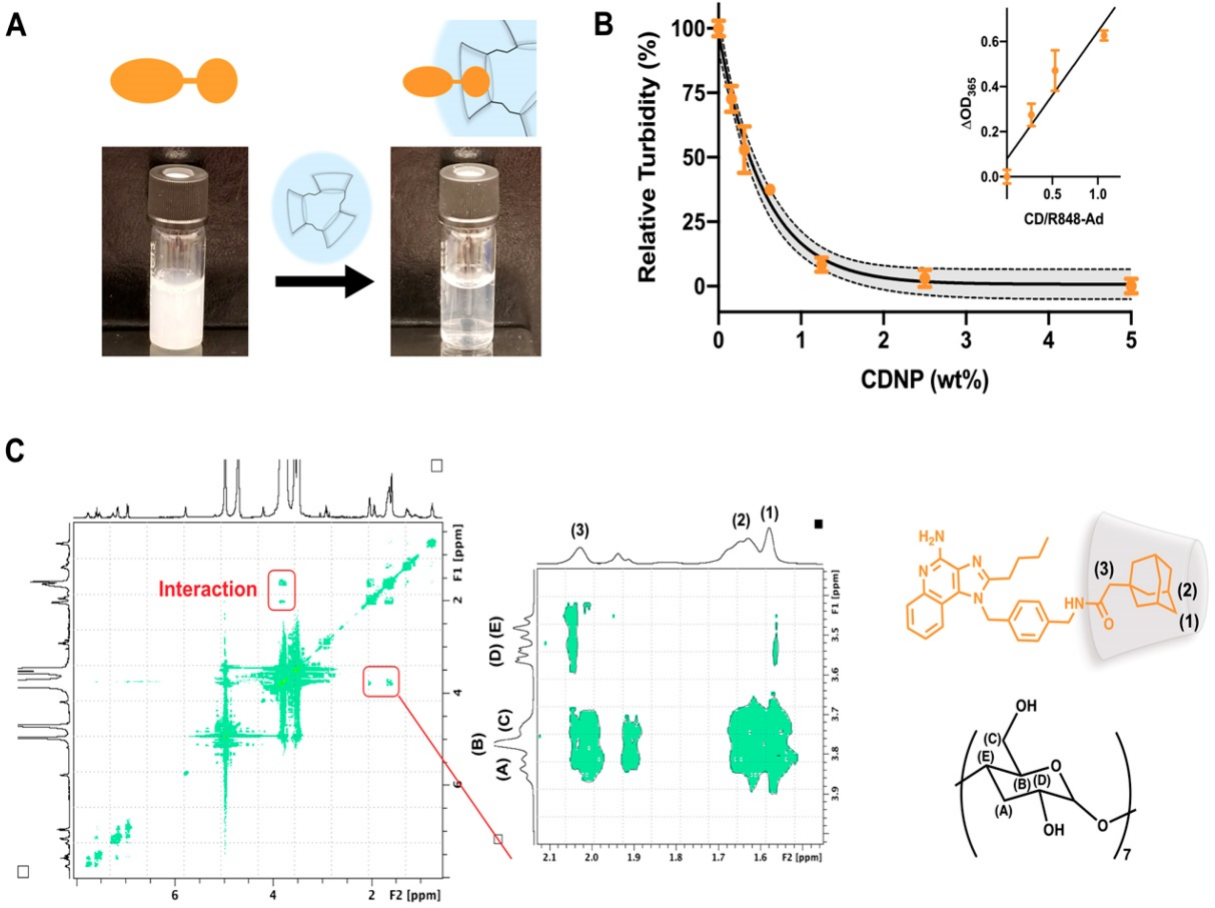
** Drug-nanoparticle interaction through adamantane. (A)** Macroscopic images of R848-Ad turbidity in aqueous solution (left) and solubilization by addition of CDNP (right). (**B**) Turbidity (OD, λ = 365 nm) measurement of R848-Ad solubilized by CDNP. Black line: exponential decay ± 95% CI (shaded). Inset: Mole ratio plots showing the change in turbidity as a function of the host-guest ratio. Black line: linear fit. Mean ± s.d.; N=3. (**C**) ROESY spectra of cyclodextrin interaction with R848-Ad, acquired in D_2_O. The expanded section highlights the specific interaction of the alkyl adamantane (orange structure) protons (1, 2) and adjacent ethyl linker (3) with the hydrophobic protons of the CD (black structure) cavity (A,B).

Drug solubilization by cyclodextrins is a common method for pharmaceutical formulation 43, made possible by the known biocompatibility of cyclodextrins which has perpetuated their use in drug delivery systems 25, 26, 44. To further examine the ability of CDNP to encapsulate R848-Ad through guest-host interaction, we examined drug solubility and intermolecular interactions. R848-Ad was insoluble in water, as indicated by high sample turbidity. However, the introduction of increasing CDNP concentrations readily solubilized the drug through nanoparticle encapsulation (Figure 4A,B), with solubility asymptotically approaching a maximum at a mole ratio of greater than 1:1 CD/Ad. Mole ratio plots were highly linear (R^2^ = 0.88) below a unity ratio, indicative of the known 1:1 interaction of Ad and CD 45, 46. The specific intermolecular geometry of the interaction was further probed by ^1^H-^1^H ROESY NMR (Figure 4C). The hydrophobic cyclodextrin cavity interacted through space with the 3-coordinated carbons, 2-coordinated bridging carbons, and the ethyl linker of the adamantane functionality, indicating formation of a deep inclusion complex specific to adamantane and not the R848 core structure. In sum, the adamantane functionality interacts specifically with the cyclodextrin nanoparticle through strong hydrophobic guest-host association, enabling high affinity drug nanoparticle interaction. Increased drug-nanoparticle affinity contributed to the prevention of drug burst release and prolonged drug release *in vitro* (Figure S4).

**Therapeutic efficacy.** Having shown that the developed drug, R848-Ad, elicits TAM polarization *in vitro* and that adamantane functionality dominates the drug interaction with CDNPs, we next sought to examine the therapeutic efficacy of the supramolecular delivery system. *In vitro* assays were initially examined to ensure that nanoformulations were non-cytotoxic and retained macrophage polarizing activity (Figure S5, S6). CDNP, R848-Ad, and the nanoformulation were not detrimental to cell viability, even at supraphysiological concentrations. Reduced polarization response was only at CDNP concentrations far exceeding those necessary for drug solubilization, potentially indicating sequestration of the drug away from the target receptor and within the nanogels. Interestingly, encapsulation of R848-Ad at moderate CDNP concentrations increased cell response, indicative of improved drug bioavailability. Such effects may be due to the endosomal location of the target TLR7/8 receptors and demonstrated endosome-associated distribution of the CDNP by concurrent anti-LAMP1 staining (Figure S3).

**Figure 5:**
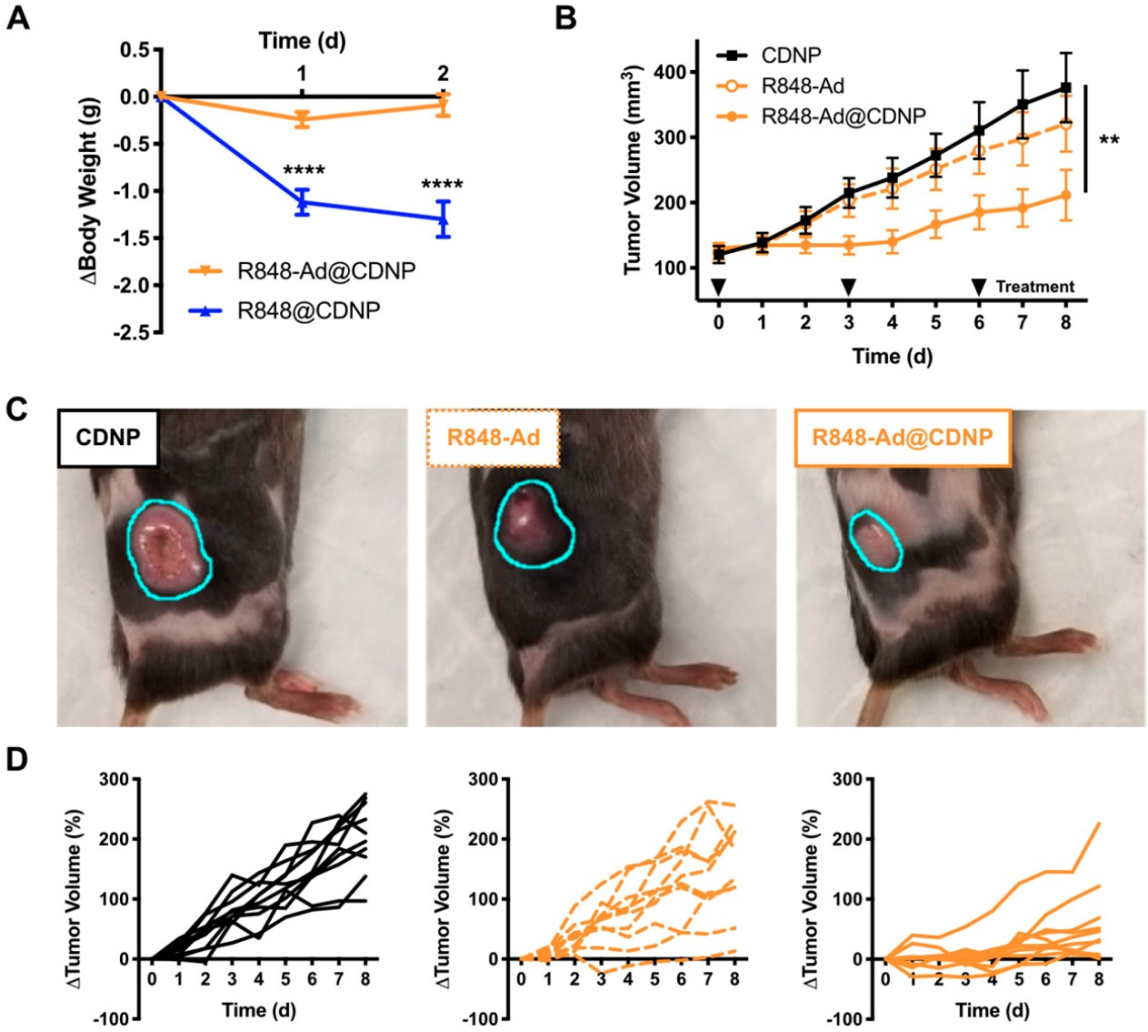
***In vivo* efficacy. (A)** Change in body weight of mice following treatment at day 0 with R848@CDNP (blue) or R848-Ad@CDNP (orange). Mean ± s.e.m; N=10; ****Tukey P<0.0001.**(B)** MC38 tumor growth curves. Mean ± s.e.m; N=10; **P=0.008 (Friedman, Dunn's multiple comparison) relative to vehicle control.** (C)** Macroscopic images of tumors at day 6 following initiation of treatment. Tumor margins are outlined (cyan) for clarity. **(D)** Individual MC38 tumor growth curves in response to treatment by CDNP vehicle controls (solid black, left), R848-Ad (dashed orange, middle), or R848-Ad@CDNP (solid orange, right).

As previously discussed, systemic administration of TLR agonists has been limited in a clinical setting by low grade adverse events (headache, fever). We expected that inhibition of side effects may be possible through drug loading in nanoparticles, as has been previously shown for chemotherapeutic agents 47. Thus, we directly compared nanoparticles that were loaded with either R848 (R848@CDNP) or R848-Ad (R848-Ad@CDNP) administered in C57BL/6 mice by intravenous (i.v.) injection at equivalent molar dosing; administration of the free drug compounds was not examined due to the known propensity for rapid weight loss to occur with delivery of unencapsulated R848 at these doses. Rapid and progressive weight loss, indicative of off-target drug effects and undesired systemic inflammatory response 48, occurred in mice receiving R848@CDNP (Figure 5A). Weight loss was dose limiting, preventing the further examination of repetitive dosing in subsequent tumor growth models. On the contrary, R848-Ad@CDNP treated mice maintained body weight relative to baseline values.

As R848-Ad@CDNP was well tolerated *in vivo*, we next examined the effects of repetitive systemic administration. In mice bearing MC38 tumors, cohorts treated by either vehicle control (CDNP) or free drug (R848-Ad) had similar progressive tumor growth (P=0.2, Dunn's test). By contrast, R848-Ad@CDNP treated mice showed a reduced rate of tumor growth (P=0.002, Dunn's test) and noticeably smaller tumors than other cohorts (Figure 5B,C). Differences in tumor growth were all the more apparent when considering heterogeneity of tumor response (Figure 5D). Dosing by free drug did moderately attenuate tumor growth and resulted in regression of 1/10 tumors, indicative of *in vivo* drug efficacy. However, efficient delivery of the drug by CDNP perpetuated a homogenization of response with the majority of mice showing attenuated tumor growth and reduction in tumor size (5/10) at maximum response.

While complete toxicity studies are an important topic for continued study, therapy by R848-Ad@CDNP was well tolerated. Systemic delivery of immune agonists, including TLR7/8 agonists, typically results in interferon response, flu like symptoms, and concurrent weight loss 49, 50. Here, body weight is thus considered as a metric for this undesirable and dose-limiting side effect. During the course of repetitive treatment, only moderate weight loss (<5.0%, Supplementary Figure S7) was observed with recovery of weight gain later in the course of study. In sum, these studies demonstrate the potential of optimized drug candidates to effectively induce anti-cancer immune activity while limiting systemic side effects which have hampered clinical translation.

## Conclusion

Tumor-associated macrophages play a central role in the establishment and maintenance of the tumor immune microenvironment. As such, they have been increasingly investigated as a therapeutic target, including through macrophage depletion or re-education from a tumor supportive (M2-like) to a tumor destructive (M1-like) phenotype. Of these methods, re-education is arguably the more powerful approach, as it directly combats the tumor through acquisition of an immune supportive environment. In this study, we sought to leverage the strong supramolecular association of adamantane and cyclodextrin to achieve efficient loading of TAM polarizing agents into cyclodextrin nanoparticles. Through iterative synthesis starting with a highly potent TLR7/8 agonist, we developed an adamantane modified drug which retained activity against the target receptors and which was incorporated into the nanoparticle by simple mixing in aqueous conditions. The delivery of such TLR agonists is known to aid in the development of an immunoresponsive tumor microenvironment, likely through a combination of direct effects on macrophages, and cooperation with other cell types which leads to the development of adaptive immune response through indirect effects 5, 51-53. *In vivo*, the supramolecular association limited systemic toxicity as compared to the unmodified TLR7/8 agonist while arresting tumor growth. Macrophage targeted therapeutics are poised to revolutionize the landscape of cancer treatment, either alone or in combination with standard of care checkpoint therapies 10, 54, 55. Our data support the continued development of optimized TAM targeted therapeutic drug delivery systems to achieve clinical success.

## Supplementary Material

Supplementary methods and figures.

## Abbreviations

Ad: adamantane
BMDM: bone marrow derived macrophage
CD: β-cyclodextrin
CDNP: cyclodextrin nanoparticle
DAMP: danger-associated molecular pattern
EDC: (1-ethyl-3-(3-dimethylaminopropyl)carbodiimide hydrochloride)
MES: 2-(N-morpholino)ethanesulfonic acid
MWCO: molecular weight cut-off
NHS: N-Hydroxysuccinimide
PAMP: pathogen-associated molecular pattern
PDI: polydispersity index
R848: resiquimod
R848-Ad: adamantane-conjugated resiquimod
TAM: tumor-associated macrophage
TIME: tumor immune microenvironment
TLR: toll-like receptor
