# Supplementary material for: Development of Adamantane-Conjugated TLR7/8 Agonists for Supramolecular Delivery and Cancer Immunotherapy
Source: Theranostics. 2019 Nov 26;9(26):8426–36. doi: 10.7150/thno.35434 (PMC6924259; doi:10.7150/thno.35434)
Supplement: Supplementary file 1 — Supplementary methods and figures. [file thnov09p8426s1.pdf]

Supporting Information

Development of Adamantane-Conjugated TLR7/8 Agonists for Supramolecular Delivery and Cancer Immunotherapy

Christopher B. Rodell[1], Maaz S. Ahmed[1], Christopher S. Garris[1,2], Mikael J. Pittet[1], Ralph Weissleder[1,3,4,*]

[1] Center for Systems Biology, Massachusetts General Hospital, Boston, MA 02114

[2] Graduate Program in Immunology, Harvard Medical School, Boston, MA 02115

[3] Department of Radiology, Massachusetts General Hospital, Boston, MA 02114

[4] Department of Systems Biology, Harvard Medical School, Boston, MA 02115

*R. Weissleder, MD, PhD
Center for Systems Biology
Massachusetts General Hospital
185 Cambridge St, CPZN 5206
Boston, MA, 02114
617-726-8226
ralph_weissleder@hms.harvard.edu

Supplementary Methods

General Considerations. All commercially available compounds were purchased and used as received. Synthesis of 2-(4-amino-2-ethoxymethyl-1H-imidazo{4,5-c}quinolin-1-yl)ethaneamine (Compound **A**) was completed following previously reported synthetic route [1, 2]. Synthesis of 1-(4-aminobutyl)-2-(ethoxymethyl)-1H-imidazo{4,5-c}quinolin4-amine (Compound **B**) was completed following previously reported synthetic route [2, 3]. 1-(4-(Aminomethyl)benzyl)-2-butyl-1H-imidazo{4,5-c}quinolin-4-ylamine (Compound **C**) was obtained from R&S Chemicals, Inc. (Kannapolis, NC). Reaction mixtures were purified using a Biotage® SNAP Bio C18 300 Å 10 g on a Biotage® Isolera with a gradient composed of water (0.1% formic acid) and acetonitrile (0.1% formic acid) for reversed-phase chromatography. P1 is a normal phase column chromatography solvent freshly prepared mixture composed of water, methanol, and acetonitrile mixed in a 1:1:1 ratio. A note of caution on the use of P1, this solvent mixture should never be stored for prolonged periods of time as the decomposition of acetonitrile to acetamide is catalyzed by water/methanol. ^{1}H and ^{13}C NMR spectra were recorded on a Bruker AC-400 MHz spectrometer. ESI mass spectra were recorded using a Waters 3100 Mass Detector.

Synthesis of 2. 1-Adamantaneacetic acid (124 mg, 0.69 mmol), EDC (147 mg, 0.950 mmol), NHS (79 mg, 0.69 mmol) were dissolved in DMF (5 mL) with *N,N*-diisopropylethylamine (2 mL). To this Compound **A** (201 mg, 0.63 mmol) was added and the reaction was allowed to proceed for 20 minutes at room temperature. After which, the reaction was concentrated under reduced pressure. The crude reaction mixture was purified via reversed-phase chromatography. Fraction of similar purity were combined and concentrated to yield the desired product **2** (9 mg, 3 % yield). MS (ESI) calculated for $C_{27}H_{35}N_5O_2$, m/z 461.28, found 462 $(M + H)^+$.

Synthesis of 3. 1-Adamantaneacetic acid (13.6 mg, 0.07 mmol) and and HOBt (9.5 mg, 0.07 mmol) were dissolved in DMF (1.0 mL) with *N,N*-diisopropylethylamine (0.25 mL). To this resiquimod (20 mg, 0.06 mmol, SelleckChem) in DMF (1 mL) was added and the reaction was allowed to proceed for 24 hours at 50 C. After which, the reaction was concentrated under reduced pressure. The crude reaction mixture was purified via normal-phase chromatography starting with EtOAc with a gradient increasing to completely P1. Fraction of similar purity were combined and concentrated to yield the desired product **3** (4.5 mg, 13 % yield). MS (ESI) calculated for $C_{29}H_{38}N_4O_3$, m/z 490.29, found 491 $(M + H)^+$.

Synthesis of 4. Compound **A** (131.9 mg, 0.46 mmol) was dissolved in DMF (2 mL) with *N,N*-diisopropylethylamine (0.20 mL) to this was added Azido-PEG$_5$-NHS ester (100 mg, 0.23 mmol, Santa Cruz Biotechnology). The reaction was allowed to proceed for 4 h at room temperature. After which, the reaction was concentrated under reduced pressure. The crude reaction mixture

was purified via reversed-phase chromatography. Fraction of similar purity were combined and concentrated to yield the desired intermediate product (45.3 mg, 32.6 % yield). The intermediate (14.7 mg, 0.024 mmol) and 1-adamantylacetylene (4.3 mg, 0.027 mmol, Matrix Scientific) were dissolved in 12 mL of 1:1 mixture of water:t-butanol. To this a solution of sodium ascorbate and copper sulfate was added, prepared by the mixing of 2.4 µL of a freshly prepared solution of sodium ascorbate (10 mg in 50 µL) with 2.4 µL of a freshly prepared solution of copper sulfate (10 mg in 50 µL). The reaction was allowed to react for 16 hours with vigorous mixing. The crude reaction mixture was purified via reversed-phase chromatography. Fraction of similar purity were combined and concentrated to yield the desired product **4** (7.9 mg, 43 % yield). MS (ESI) calculated $C_{40}H_{58}N_8O_7$, m/z 762.44, found 763 $(M + H)^+$.

Synthesis of 5. Compound **A** (144 mg, 0.51 mmol) was dissolved in DMF (5 mL) with *N,N*-diisopropylethylamine (2 mL) to this was added Azido-PEG$_2$-NHS ester (81.4 mg, 0.40 mmol, Santa Cruz Biotechnology). The reaction was allowed to proceed for 4 h at room temperature. After which, the reaction was concentrated under reduced pressure. The crude reaction mixture was purified via reversed-phase chromatography. Fraction of similar purity were combined and concentrated to yield the desired intermediate product (188 mg, 99% yield). The intermediate (188 mg, 0.40 mmol) and 1-adamantylacetylene (70.4 mg, 0.44 mmol, Matrix Scientific) were dissolved in 50 mL of 1:1 mixture of water:t-butanol. To this a solution of sodium ascorbate and copper sulfate was added, prepared by the mixing of 25 µL of a freshly prepared solution of sodium ascorbate (10 mg in 50 µL) with 25 µL of a freshly prepared solution of copper sulfate (10 mg in 50 µL). The reaction was refluxed for 4 hours with vigorous mixing. The crude reaction mixture was purified via reversed-phase chromatography. Fraction of similar purity were combined and concentrated to yield the desired product 5 (5.6 mg, 2 % yield). MS (ESI) calculated $C_{34}H_{46}N_8O_4$, m/z 630.36, found 632 $(M + H)^+$.

Synthesis of 6. Compound **A** (148 mg, 0.520 mmol) was dissolved in acetic anhydride (86.5 mL) with *N,N*-diisopropylethylamine (0.174 mL). The reaction was allowed to proceed for 30 minutes at room temperature. After which, the reaction was concentrated under reduced pressure to yield the desired product 6 (169.9 mg, 99 % yield). MS (ESI) calculated $C_{17}H_{21}N_5O_2$, m/z 327.17, found 328 $(M + H)^+$.

Synthesis of 7. Compound **B** (26.4 mg, 0.100 mmol) was dissolved in acetic anhydride (0.100 mL) with *N,N*-diisopropylethylamine (0.850 mL). The reaction was allowed to proceed for 30 minutes at room temperature. After which, the reaction was concentrated under reduced pressure to yield the desired product **7** (29.9 mg, 84% yield). MS (ESI) calculated $C_{19}H_{25}N_5O_2$, m/z 355.20, found 356 $(M + H)^+$.

Synthesis of 9. Compound **C** (5.6 mg, 0.02 mmol) was dissolved in acetic anhydride (0.1 mL) with *N,N*-diisopropylethylamine (0.1 mL). The reaction was allowed to proceed for 30 minutes at room temperature. After which, the reaction was concentrated under reduced pressure to yield the desired product **9** (6.2 mg, 99 % yield). MS (ESI) calculated for $C_{24}H_{27}N_5O$, m/z 401.22, found 402 $(M + H)^+$.

Synthesis of 10. 1-Adamantaneacetic acid (28 mg, 0.14 mmol), HATU (53 mg, 0.14 mmol), and NHS (17 mg, 0.14 mmol) was dissolved in DMF (5 mL) with *N,N*-diisopropylethylamine (0.1 mL) under Ar. After 30 mins, Compound **C** (40 mg, 0.11 mmol) dissolved in minimal DMF and were added and the reaction was allowed to proceed for 30 minutes at room temperature. After which, the reaction was concentrated under reduced pressure. The crude reaction mixture was purified via reversed-phase chromatography. Fraction of similar purity were combined and concentrated to yield the desired product **10** (59 mg, 78 % yield). {**10**+formic acid} ^{1}H NMR (400 MHz, $CDCl_3$) δ 8.73 (s, 1H), 7.72 (d, J = 8.3 Hz, 1H), 7.56 (d, J = 8.3 Hz, 1H), 7.33 (t, J = 7.8 Hz, 1H), 7.19 (d, J = 7.7 Hz, 3H), 7.08 (t, J = 7.7 Hz, 1H), 6.90 (d, J = 7.8 Hz, 2H), 5.91 (d, J = 5.9 Hz, 1H), 5.63 (s, 2H), 4.31 (d, J = 5.8 Hz, 2H), 2.78 (t, J = 7.8 Hz, 2H), 1.86 (s, 2H), 1.71 (p, J = 7.8 Hz, 2H), 1.59 (d, J = 12.4 Hz, 4H), 1.51 (d, J = 2.9 Hz, 10H), 1.34 (p, J = 7.5 Hz, 2H), 1.18 (s, 3H), 0.85 (t, J = 7.3 Hz, 3H). ^{13}C NMR (101 MHz, $CDCl_3$) δ 171.11, 171.00, 155.84, 150.59, 139.29, 137.58, 134.89, 133.58, 128.93, 128.69, 127.85, 125.74, 125.59, 124.03, 121.48, 120.32, 114.00, 113.26, 51.68, 48.90, 43.00, 42.71, 36.80, 34.05, 32.95, 29.80, 29.60, 29.39, 28.68, 27.10, 25.72, 25.05, 22.79, 22.53, 13.84. MS (ESI) calculated for $C_{34}H_{41}N_5O$, m/z 535.74, found 537 $(M + H)^+$. Purity was determined to be >95% by both 1H NMR and HPLC analysis (Figure S8).

Visualization of drug binding. Visualization of R848 derivative interactions with TLR7 (protein databank number: 5gmh) was performed with the aid of PyMOL software package [4]. The crystal structure of monkey TLR7-R848 was chosen as a surrogate for the human receptor due to the high quality data available and conservation of protein structure across species. TLR7 exists *in vivo* as a dimeric species; however, the PDB data file used for drug optimization is a monomer projection and requires workup to display the dimeric component prior to analysis.

Supplementary Data

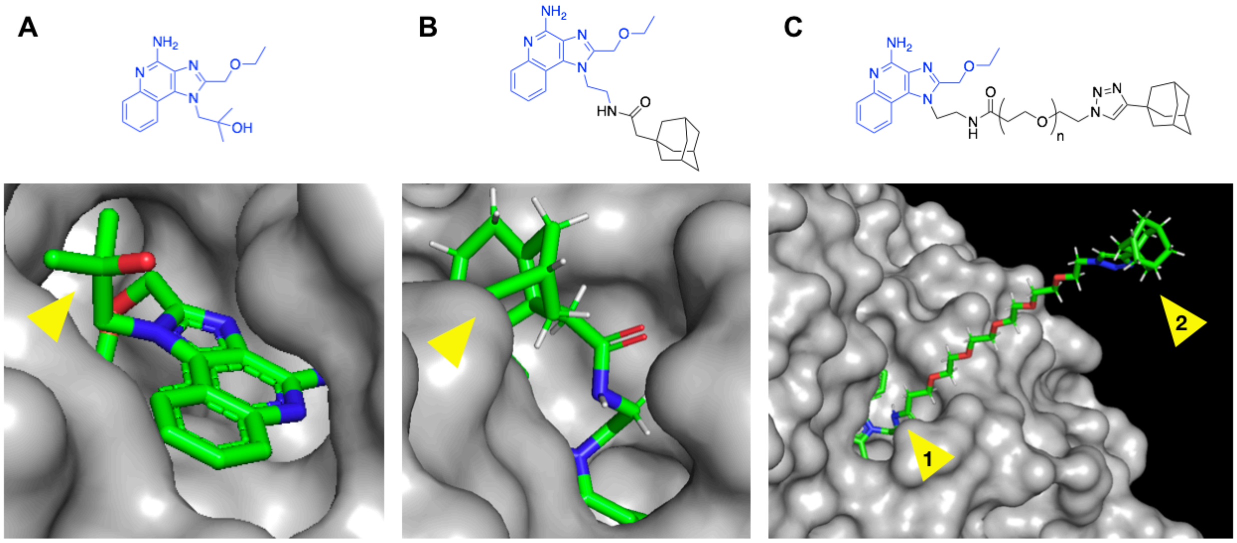

Figure S1. Crystal structure of R848 derivative binding. (**A**) For R848, the tertbutyl tail is cleared from contact with the native protein structure. (**B**) Following direct modification by adamantane (Generation 1, compound **(2)**), drug accessibility of the binding site is statically hindered (indicated: yellow arrow). (**C**) The incorporation of suitable linkers, such as PEO_5 shown (Generation 2, compound **(5)**), enable access of the R848 core structure to the binding site (indicated: yellow arrow, 1) while displacing adamantane to a less sterically hindering location (indicated: yellow arrow, 2).

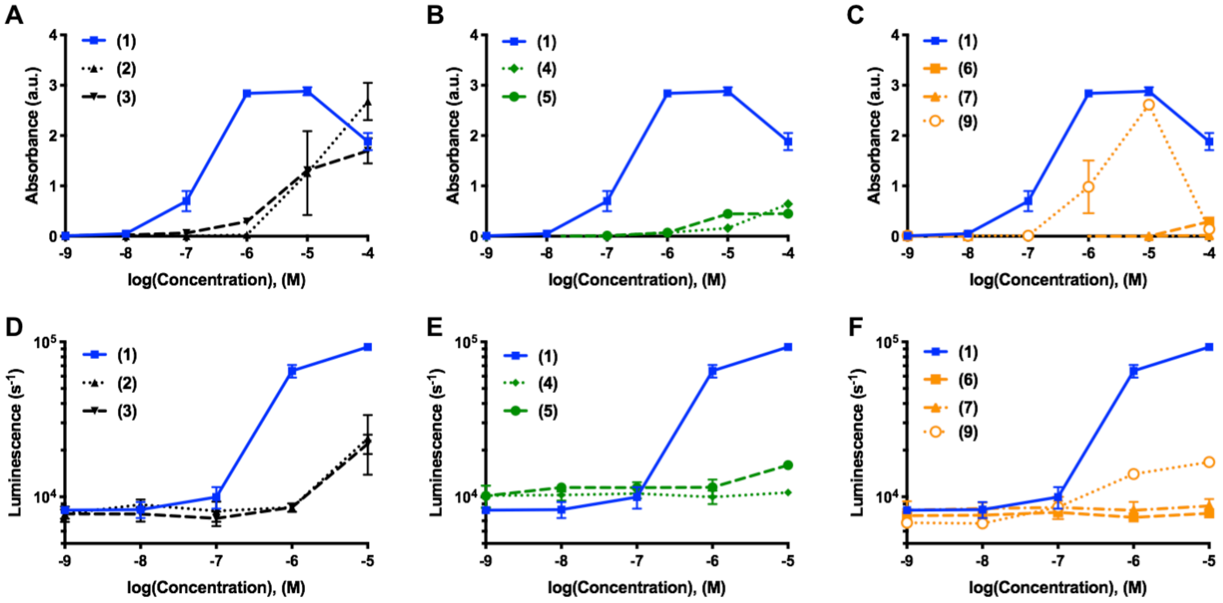

Figure S2. Screening results versus human receptor & cell line. Concentration-dependent activity of compounds in Generation 1 (**A**,**D**: black), Generation 2 (**B**,**E**: green), and Generation 3 (**C**,**F**, orange) assayed against HEK-Blue hTLR8 (top) and THP1-Lucia NFkB (bottom) reporter cell lines. Results represent the mean ± s.d.; N=4.

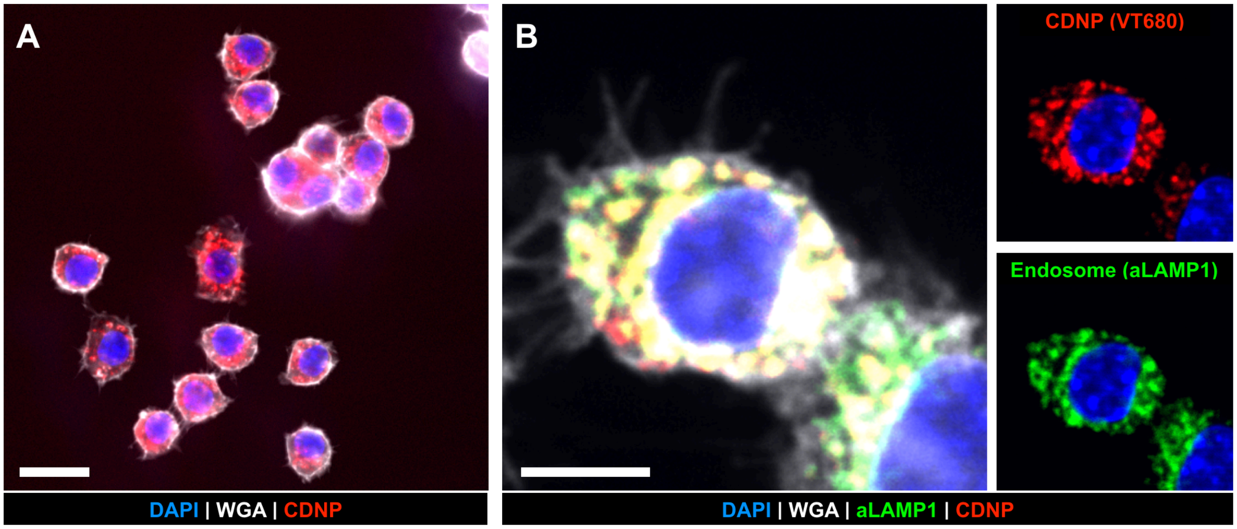

Figure S3. Cellular biodistribution of CDNP-VT680. The uptake of VivoTag 680 labeled CDNP (CDNP-VT680) was examined in RAW264.7 cells. (**A**) Fluorescence images show punctate accumulation of CDNP within cells within 1 hr. Staining: DAPI (nuclei, blue); WGA-AF488 (cell membrane, white); CDNP-VT680 (nanoparticle, red). Scale bar: 10 μm. (**B**) Confocal fluorescence microscopy highlights lysosomal accumulation of the nanoparticle anticipated to improve delivery to endosomal TLR receptors. Staining: DAPI (nuclei, blue); WGA-AF555 (cell membrane, white); anti-LAMP1-AF488 (lysosome, green); CDNP-VT680 (nanoparticle, red). Scale bar: 5 μm.

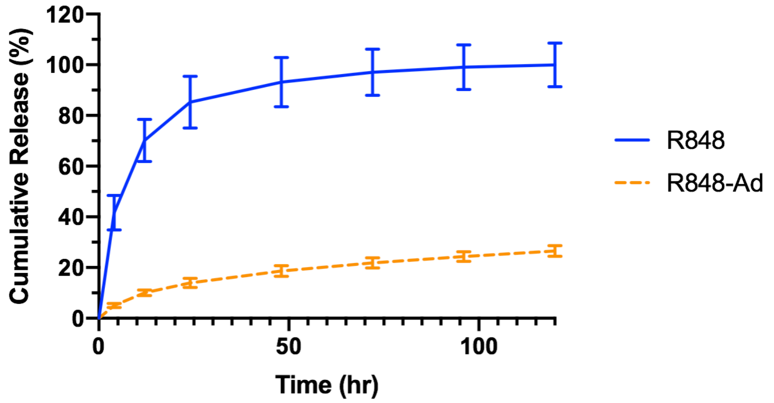

Figure S4. Drug release profiles. Cumulative release of R848 and R848-Ad, investigated in an equilibrium dialysis setup at 37 C. Results represent the mean ± s.d.; N=3 independent samples.

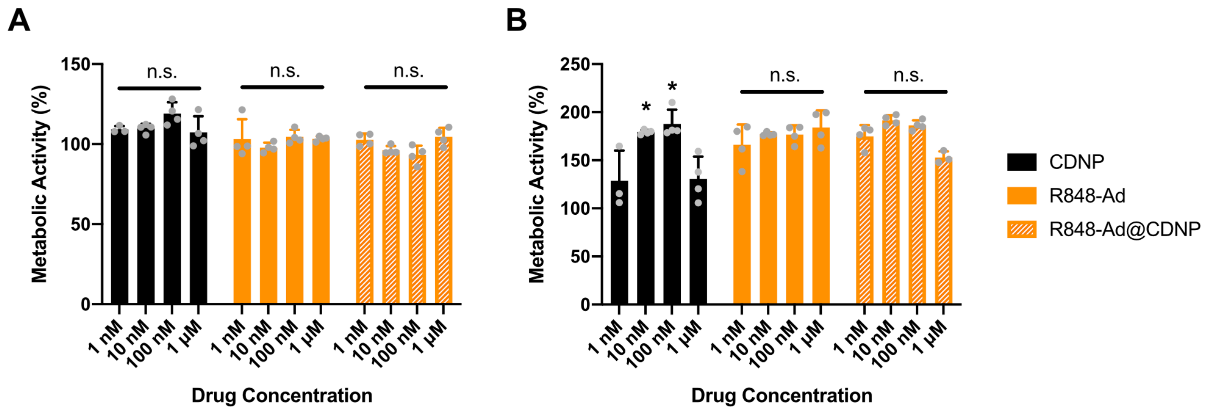

Figure S5. Metabolic assays. The metabolic activity of MC38 cells (**A**) RAW264.7 cells (**B**) was assayed by PrestoBlue 48 hr after treatment by CDNP, R848-Ad, and R848-Ad@CDNP. No concentration dependent loss of metabolic activity indicative of cytotoxicity was observed. Data represent mean ± s.d.; N=4, normalized to non-treated controls; *P<0.5, two-way ANOVA with post hoc Tukeys HSD.

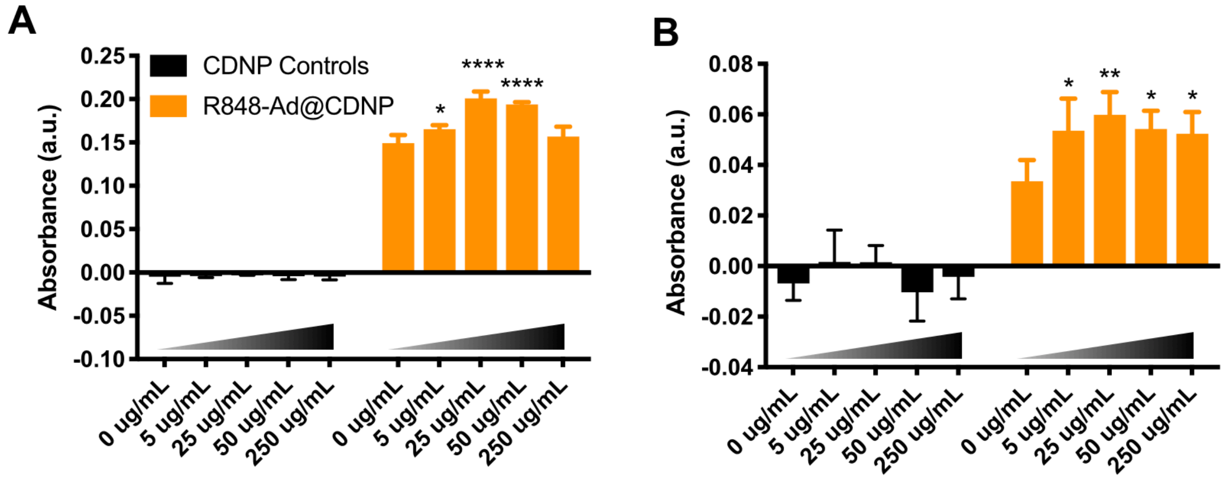

Figure S6. CDNP enhances R848-Ad drug activity *in vitro*. The activity of R848-Ad@CDNP was examined in HEK-Blue mTLR7 (**A**) and HEK-Blue hTLR8 (**B**) cells at a fixed concentration of 10 nM R848-Ad and an increasing CDNP concentration (0-250 µg/mL). CDNP controls (black, left) did not activate TLR7 or TLR8 activity. CDNP increased R848-Ad activity, relative to the free drug. Increased activity was attenuated at high CDNP concentrations (molar ratio CD/Ad >> 1). Results represent the mean ± s.d. of the absorbance at = 662 nm; N=4; ****p<0.0001, ***p<0.001, **p<0.01, *p<0.01 vs free drug (0 µg/mL CDNP).

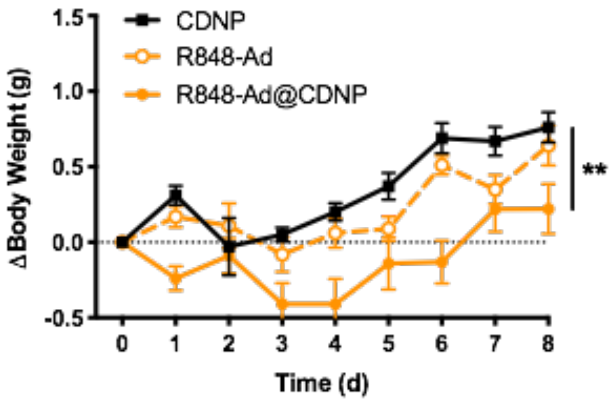

Figure S7. Change in body weight of mice following treatment at day 0, 3, and 6 with CDNP vehicle controls (solid black, left), R848-Ad (dashed orange, middle), or R848-Ad@CDNP (solid orange, right). Mean ± s.e.m; N = 10; **P < 0.01 (Friedman, Dunn's multiple comparison) relative to vehicle control.

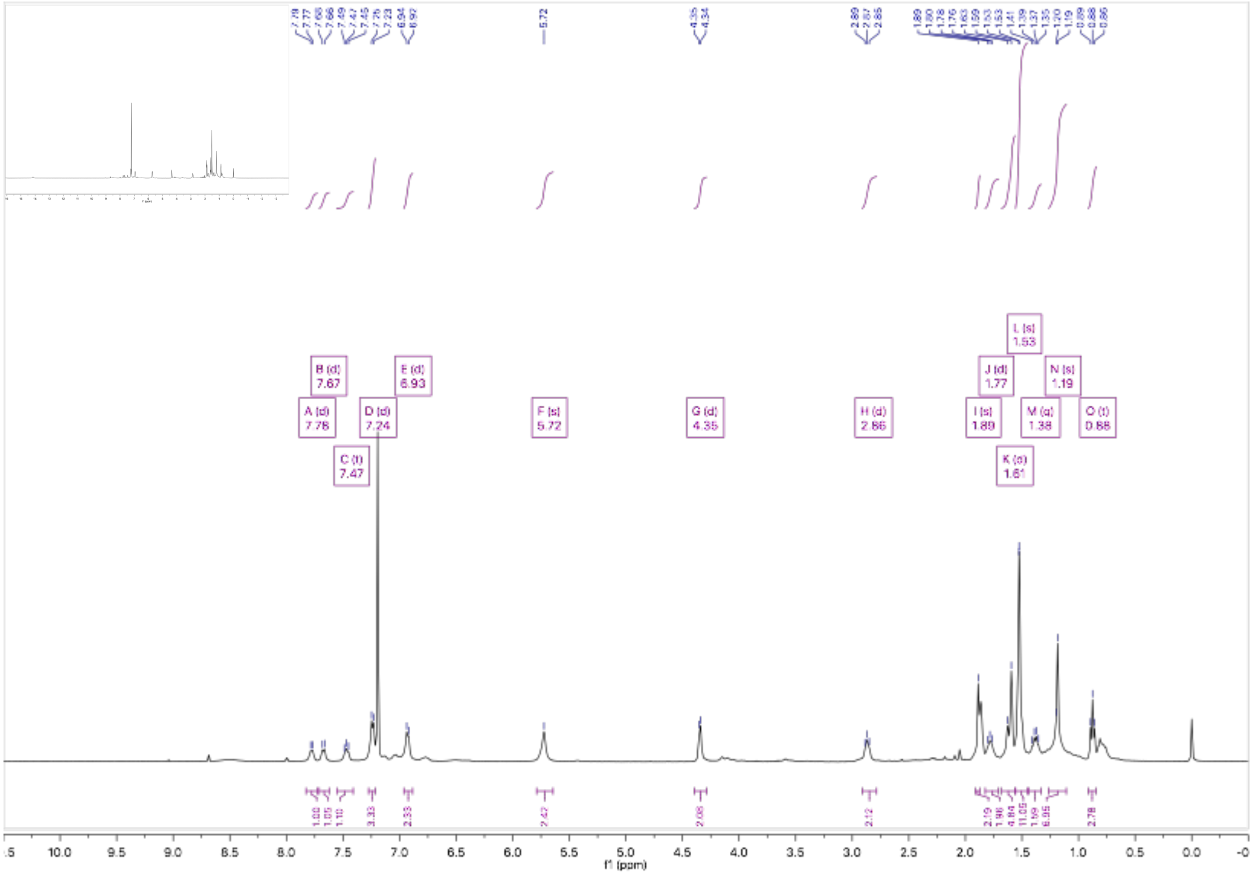

Figure S8. 1H NMR spectra of R848-Ad. The final product, R848-Ad, was determined via ^{1}H NMR and HPLC (at both 250 and 315 nm) to be spectroscopically pure (>95%).

Supplementary References

1. Griesgraber GW. Sulfonamide and sulfamide substituted imidazoquinolines. 2004; U.S. Patent No. 6,331,539.
2. Gerster JF, Lindstrom KJ, Miller RL, Tomai MA, Birmachu W, Bomersine SN et al. Synthesis and structure− activity-relationships of 1 H-imidazo [4, 5-c] quinolines that induce interferon production. J Med Chem. 2005; 48: 3481-3491.
3. Fisher K, Laga R, Lynn G, Seymour L, Seder R. Polymer adjuvant. 2017; U.S. Patent Application No. 15/517,121.
4. Zhang Z, Ohto U, Shibata T, Krayukhina E, Taoka M, Yamauchi Y et al. Structural analysis reveals that Toll-like receptor 7 is a dual receptor for guanosine and single-stranded RNA. Immunity. 2016; 45: 737-748.
